# Decreased TREC and KREC levels in newborns with trisomy 21

**DOI:** 10.3389/fped.2024.1468635

**Published:** 2024-10-17

**Authors:** Andrey Marakhonov, Anna Mukhina, Elena Vlasova, Irina Efimova, Natalya Balinova, Yulia Rodina, Dmitry Pershin, Zhanna Markova, Marina Minzhenkova, Nadezhda Shilova, Dzhaina Mudaeva, Djamila Saydaeva, Taisiya Irbaieva, Svetlana Matulevich, Elena Belyashova, Grigoriy Yakubovskiy, Inna Tebieva, Yulia Gabisova, Murat Ikaev, Nataliya Irinina, Liya Nurgalieva, Elena Saifullina, Tatiana Belyaeva, Olga Romanova, Sergey Voronin, Rena Zinchenko, Anna Shcherbina, Sergey Kutsev

**Affiliations:** ^1^ Research Centre for Medical Genetics, Moscow, Russia; ^2^ Oncology and Immunology, Dmitry Rogachev National Medical Research Center of Pediatric Hematology, Moscow, Russia; ^3^ Regional Children’s Clinical Hospital No. 1, Yekaterinburg, Russia; ^4^ Republican Perinatal Center, Grozny, Russia; ^5^ State Budgetary Institution “Maternity Hospital” of the Ministry of Healthcare of the Chechen Republic, Grozny, Russia; ^6^ Department of Maternity and Childhood, Ministry of Health Chechen Republic, Grozny, Russia; ^7^ S.V. Ochapovsky Regional Clinical Hospital №1, Krasnodar, Russia; ^8^ Orenburg Regional Clinical Hospital No. 2, Orenburg, Russia; ^9^ Ryazan Regional Clinical Perinatal Center, Ryazan, Russia; ^10^ North-Ossetian State Medical Academy, Vladikavkaz, Russia; ^11^ Republican Childrens Clinical Hospital of the Republic of North Ossetia-Alania, Vladikavkaz, Russia; ^12^ The State Budgetary Healthcare Institution of the Vladimir Region “Regional Clinical Hospital”, Vladimir, Russia; ^13^ Republican Center for Medical Genetics, Ufa, Russia; ^14^ Bashkir State Medical University, Ufa, Russia; ^15^ Clinical Diagnostic Center “Maternal and Child Health”, Yekaterinburg, Russia

**Keywords:** newborn screening, TREC, KREC, lymphopenia, Down syndrome, trisomy 21

## Abstract

Newborn screening (NBS) for severe combined immunodeficiency (SCID) has been widely implemented to enable early detection and intervention. Trisomy 21, commonly known as Down syndrome (DS), poses unique challenges in NBS due to its frequent association with T and/or B cell lymphopenia. The pilot NBS screening program recently conducted in Russia was aimed to identify both severe T and B cell deficiencies by measuring TREC and KREC. This study aims to evaluate the incidence of DS in newborns who participated in the pilot program, assess their TREC and KREC values, and determine the proportion of DS newborns potentially identifiable through T/B lymphopenia NBS. We conducted a retrospective analysis of the data obtained during the pilot NBS program, involving 202,908 newborns from eight regions of Russia. The study identified 157 patients with trisomy 21 among the screened cohort, resulting in a DS birth prevalence of 1:1,284. Median TREC and KREC values did not significantly differ between full-term and pre-term subgroups of DS patients. TREC values in DS newborns were decreased and comparable to those of the extremely preterm newborns. DS newborns also demonstrated significant differences in KREC values as compared to the general cohort regardless of gestational age. Our data suggests abnormalities of T- and B-cell lineages development and requires further investigation. This article highlights the need for increased awareness of the intrinsic immunological defects associated with DS. The findings underscore the importance of continued follow-up and comprehensive support by healthcare teams for individuals with DS.

## 1 Introduction

Primary immunodeficiencies (PIDs), also known as inborn errors of immunity (IEIs), encompass a spectrum of genetically determined disorders characterized by defects in various components of the immune system (1). One notable PID is severe combined immunodeficiency (SCID), typified by the absence or markedly reduced numbers of T-lymphocytes, and in certain instances, deficient or malfunctioning B- and NK-cells (2). Affected newborns with SCID typically exhibit no overt clinical symptoms at birth, but between 3 and 6 months of age, they manifest severe, recurrent infections of bacterial, viral, and fungal origins, which, in the absence of appropriate intervention, can culminate in mortality within the first one to two years of life (3). Newborn screening for SCID, involving the quantification of T-cell receptor excision circles (TRECs) and/or kappa-deleting recombination excision circles (KRECs) in dried blood spots collected on filter paper test cards, is effectively implemented in numerous countries worldwide (4). This screening facilitates the early detection of affected individuals, enhancing the prospects for successful therapeutic intervention during the presymptomatic phase of the disease (5). It is noteworthy that beyond SCID, syndromic forms of PID, such as 22q11.2 deletion syndrome, CHARGE syndrome, and trisomy 21, among others, can also be identified through newborn screening (6). The incorporation of KREC measurements into NBS for PIDs also facilitates the identification of various conditions characterized by B-cell lymphopenia, with agammaglobulinemia being the most severe form (7). This underscores the importance of comprehensive screening strategies to encompass a broad spectrum of immunodeficiency disorders, enabling early recognition and intervention to improve clinical outcomes in affected individuals.

Down syndrome (DS, OMIM #190685) is a prevalent chromosomal disorder resulting from partial or complete trisomy of chromosome 21. In the world population, DS prevalence is assessed as 1 per 700–1,000 people (8, 9). In Russia, the observed live birth prevalence is 1:1,209 which is primarily linked to the performed prenatal screening and family planning choices (10). DS is characterized by distinctive facial features, intellectual disability, and systemic involvement. Individuals with DS commonly experience neurological issues, and eye abnormalities leading to reduced visual acuity, hearing loss, congenital heart defects, pulmonary complications, renal anomalies, thyroid dysfunction, diabetes, and other symptoms. Furthermore, DS is associated with an increased risk of myeloid leukemia, growth delay, obesity, and other health conditions (11, 12). Immunological deficits are also described in DS patients (13, 14). Individuals with DS often exhibit hypoplastic thymus and a diminished number of mature thymocytes, leading to reduced T-cell populations in peripheral blood (15). Newborns with DS constitute a distinct subset of patients identified through newborn screening (NBS) for primary immunodeficiency disorders (PID) constituting up to 11% of syndromic cases identified (16). However, it remains unclear which subset of DS patients would be test-positive on NBS for T and B cell lymphopenia.

Recently, we conducted a pilot NBS program in Russia, involving the detection of TREC and KREC, encompassing a cohort of more than 202,908 newborns. Notably, the NBS program identified one patient with DS among the screened cohort (17). The objectives of the current study were to assess the incidence of DS, evaluate TREC/KREC levels in individuals with trisomy 21, and determine the proportion of newborns with DS that can be potentially identified through NBS by performing the retrospective analysis of DS patients within the cohort of infants who participated in the pilot NBS project.

## 2 Materials and methods

### 2.1 NBS pilot program

The NBS pilot program was carried out as described earlier (17). In summary, TREC/KREC-based screening was carried out from January 1, 2022, to February 19, 2023, across eight regions of the Russian Federation. These regions included the Krasnodar Territory, Vladimir, Orenburg, Ryazan, Sverdlovsk Regions, Republic of Bashkortostan, Republic of North Ossetia-Alania, and the Chechen Republic. The total cohort screened comprised 202,908 neonates. Written parental informed consent was obtained for all newborns enrolled in the pilot NBS program.

The study was conducted in accordance with the Declaration of Helsinki and approved by the Institutional Ethics Committee of the Research Centre for Medical Genetics (protocol №1/1 dated 02 March 2022).

We collected data on the newborns’ sex, gestational age, and birth weight from the eight regions participating in the study. Unfortunately, not all regions provided complete data, resulting in some gaps in the information. The male-to-female ratio was 106.8 males per 100 females. The proportion of premature infants, defined as those born on or before 37 weeks of gestation, was 5.86%, with a 95% confidence interval ranging from 5.74% to 5.99%. The median birth weight for the entire cohort was 3,360 g, with a minimum weight of 450 grams and a maximum weight of 5,500 grams. Notably, the percentages of preterm newborns and the male-to-female ratio observed in our study were consistent with the demographic data available for the entire Russian Federation during the same period.

The NBS tests were conducted utilizing the Eonis™ SCID-SMA kit (Wallac Oy, Turku, Finland) on the JANUS Extraction instrument (Perkin Elmer, Turku, Finland). Subsequently, real-time PCR analysis was performed using Applied Biosystems QuantStudio 5 Dx instruments (Thermo Fisher Scientific, Waltham, MA, USA), according to the manufacturer's guidelines and recommendations. The cut-off values for TREC and KREC were set at 100 copies per 100,000 cells for this pilot study, in accordance with the manufacturer's recommendations and published data. Newborns with values below this threshold were flagged as positive in the screening, necessitating further analysis.

Retrospective data on newborns with chromosomal abnormalities, including DS, were collected throughout the duration of the NBS pilot project in the respective regions. The diagnosis of DS was based on clinical presentation and confirmed by karyotyping at the Genetic Counseling Centers in each participating region.

### 2.2 Statistical analysis

The data collected from the study was analyzed using GraphPad Prism 8.0.1 (GraphPad Software, San Diego, California, USA). Throughout the analysis, data were presented as median with interquartile range, unless specified otherwise. Additionally, Fisher's 95% confidence interval (95%CI) for proportional data was computed using WinPepi v. 11.65 software (18).

## 3 Results

During the pilot NBS study conducted in Russia, a cohort of 202,908 newborns from eight regions was analyzed. Upon retrospective examination, our study identified 157 patients presenting with various forms of trisomy 21, encompassing both translocational and nondisjunctional forms of the condition. This yielded a birth prevalence of DS during the analyzed period of 1:1,284 (95%CI: 1:1,105–1:1,521), with prevalence rates ranging from 1:789 to 1:1,561 across different regions. However, these variations in prevalence rates between regions did not reach statistical significance. The gender distribution analysis revealed a male-to-female ratio of 69 males to 62 females, which corresponds to the ratio observed in the screened cohort (*p*-value = 0.8156, *z*-test for two proportions). 22.62% (95% CI: 18.60–41.83) of the DS patients were born at less than 37 weeks of gestation, which significantly differs from the proportion observed in the general cohort (5.86%; 95%CI: 5.74%–5.99%) (*p*-value = 6.5925 × 10^−11^, *z*-test for two proportions).

**Figure 1:**
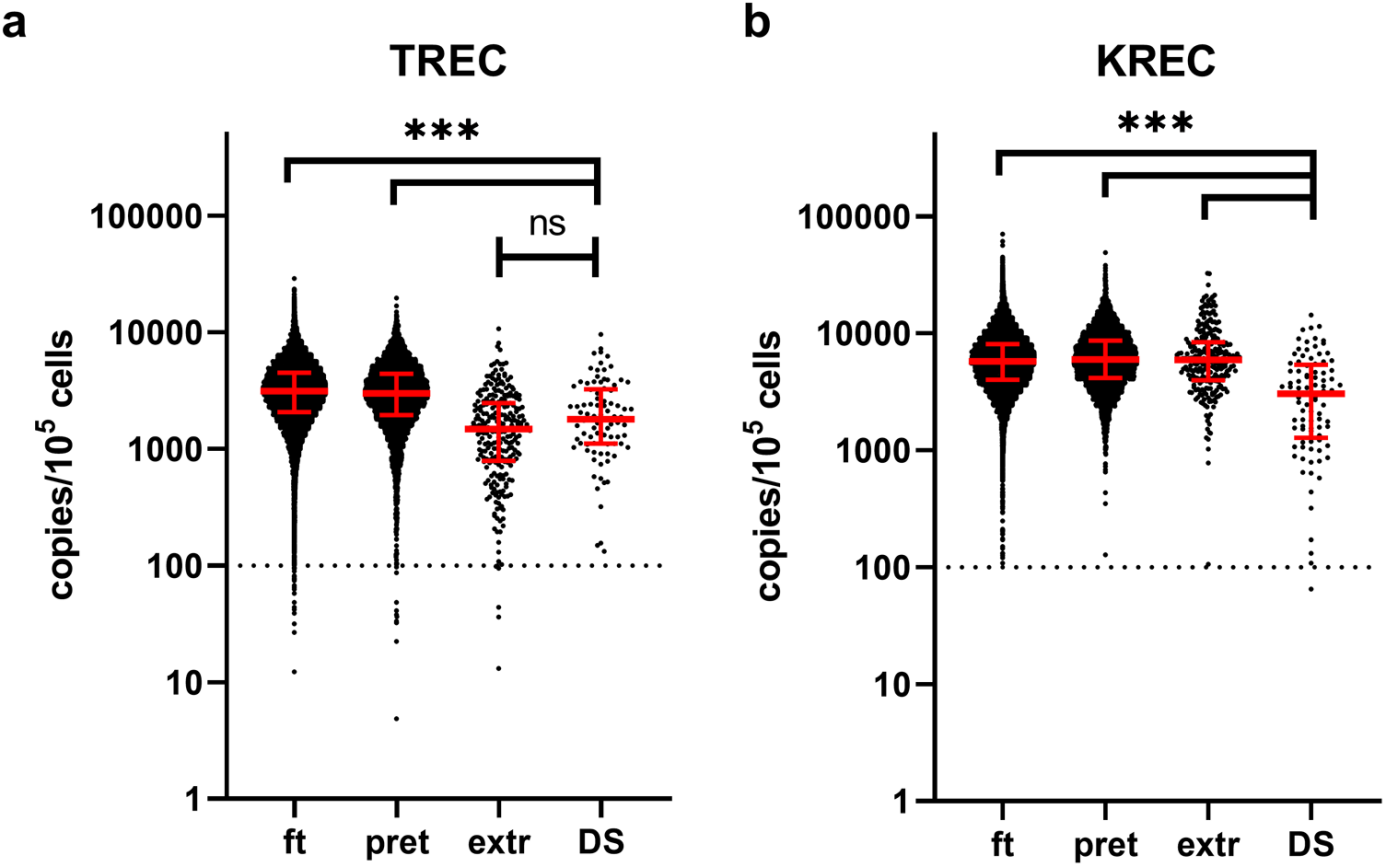
TREC **(a)** and KREC **(b)** concentrations (in copies per 10^5^ cells) in newborns of different gestational age. Red lines represent the median and interquartile range. The dotted line represents a cut-off value of concentrations of analytes (100 copies/10^5^ cells). Note the log_10_ scale on the *Y* axis. ft, full term (≥37 weeks of gestation), pret, preterm (32–37 weeks), extr, extremely preterm (<32 weeks), DS, down syndrome patients.

We subsequently analyzed the distributions of TREC and KREC values in patients with DS compared to the general cohort, stratified by different gestational ages (Figure 1, Table 1). It is worth noting that the values of TREC and KREC did not exhibit significant differences between full-term and pre-term subgroups of DS patients (*p*-value = 0.1522 and 0.1565 for TREC and KREC, respectively). As such, we opted not to stratify DS patients based on their gestational age. Our analysis revealed significantly lower median TREC values in DS newborns as compared to both full-term and preterm infants in the large cohort (*p* < 0.0001). DS TREC values were as low as such of the extremely preterm newborns (<32 weeks of gestation) (*p* = 0.0723, Dunn's multiple comparisons test). DS newborns also demonstrated dramatic differences in KREC values that were significantly lower than in the general cohort regardless of gestational age.

**Table 1:** Descriptive statistics of TREC and KREC levels (in copies per 105 cells) in newborns of different groups.

	TREC, copies/10^5^ cells				KREC, copies/10^5^ cells			
	ft	pret	extr	DS	ft	pret	extr	DS
Minimum	0.000	0.000	0.000	132.4	0.000	0.000	0.000	32.00
25% Percentile	2,072	1,956	789.2	1,114	4,006	4,157	3,975	1,281
Median	3,127	3,003	1,489	1,804	5,757	5,981	5,936	3,033
75% Percentile	4,519	4,394	2,483	3,262	8,118	8,635	8,383	5,402
Maximum	28,983	19,635	10,741	9,606	70,568	49,059	32,671	14,320
Range	28,983	19,635	10,741	9,473	70,568	49,059	32,671	14,288
5% Percentile	1,004	801.4	209.2	388.3	2,321	2,397	1,994	245.1
95% Percentile	7,273	7,120	4,422	6,443	13,328	14,178	17,506	9,883
95% CI of median								
Actual confidence level	95.00%	95.00%	95.17%	96.66%	95.00%	95.00%	95.17%	96.66%
Lower confidence limit	3,112	2,931	1,299	1,386	5,735	5,879	5,276	1,847
Upper confidence limit	3,143	3,077	1,647	2,307	5,786	6,107	6,372	3,742
Mean	3,511	3,373	1,810	2,324	6,506	6,850	7,128	3,652
Std. Deviation	2,031	2,017	1,486	1,767	3,604	3,923	4,951	2,978
Std. Error of Mean	7.265	30.19	91.61	187.3	12.90	58.73	305.3	315.7
Lower 95% CI of mean	3,497	3,314	1,630	1,952	6,481	6,735	6,527	3,025
Upper 95% CI of mean	3,525	3,432	1,990	2,697	6,531	6,965	7,729	4,280
Adjusted *P* Value (Dunn's multiple comparisons test): DS vs. groups	<0.0001	<0.0001	0.0723	*	<0.0001	<0.0001	<0.0001	*

ft, full term (≥37 weeks of gestation), pret, preterm (32–37 weeks), extr, extremely preterm (<32 weeks), DS, down syndrome patients.

* indicates a meaningless comparison within the same group.

It is noteworthy that only one child within the DS newborns group was screen-positive at NBS, with 1^st^ tier test TREC and KREC values of 253 and 32 copies/10^5^ cells, respectively. Our findings estimate the ratio of TREC/KREC screen-positive DS newborns to be 1.12% (95%CI: 0.03%–6.10%). This ratio is significantly higher than the screen-positive group in full-term newborns (0.19%, 95%CI: 0.16%–0.22%; *p*-value = 0.0414, *z*-test for two proportions) and does not differ from the screen-positive group in preterm and extremely preterm group (0.38%, 95%CI: 0.22%–0.61%, *p*-value = 0.2690; 2.66%, 95%CI: 1.08%–5.41%, *p*-value = 0.4000, respectively).

The male patient with trisomy 21 was born at 36 weeks of gestation with a weight of 2,310 g. At birth, he was diagnosed with valvular pulmonary artery stenosis without circulatory insufficiency, muscle hypotonia, and bilateral cryptorchidism.

On day 34 of life, his TREC and KREC levels at retest were low with values of 156 and 32 copies/10^5^ cells, respectively. By day 41, he exhibited low B cell counts (0.06 × 10^9^/L), while maintaining normal T-cell counts. Immunoglobulin replacement therapy and antimicrobial prophylaxis were initiated. However, his family refused to continue the treatment and further genetic testing. By the age of 24 months, he had suffered severe infections, including enterocolitis complicated by sepsis and three episodes of pneumonia, and had been diagnosed with tuberculosis of the intrathoracic lymph nodes and started on anti-tuberculosis chemotherapy. At the immunological follow-up at the age of 25 months, his CD19^+^ count was still low (0.22 × 10^9^/L) with decreased class-switched CD27 ^+^ IgM^−^IgD^−^ memory B cells (5.3%). T cell lineage was normal [CD3^+^ 2.09 × 10^9^/L, CD4^+^ 0.86 × 10^9^/L (naïve 65%), CD8^+^ 1.17 × 10^9^/L]. Serum immunoglobulin levels were normal (IgA 0.39 g/L, IgM 0.49 g/L, IgG 7.93 g/L,), without immunoglobulin substitution.

## 4 Discussion

Trisomy 21, commonly known as Down syndrome, manifests as a complex condition impacting multiple organs and systems, necessitating a comprehensive, multidisciplinary approach to follow-up and treatment (11). Trisomy 21 is associated with an elevated risk of preterm birth (<37 weeks; 22.62% vs. 5.86%), as evidenced by our study findings, which are consistent with previous research (19).

Our findings indicate that newborns with DS may show impairments in either TREC or KREC levels, regardless of gestational age, aligning with observations from other studies (13). Specifically, we found that TREC levels in DS patients are comparable to those in extremely preterm newborns. However, KREC levels in DS patients are consistently and significantly lower than those in non-DS newborns across all gestational ages. Despite this, our estimations suggest that only about 1% of DS patients could be identified through newborn screening, as they do not exhibit the profound decreases in TREC/KREC levels typically seen in conditions like SCID or agammaglobulinemia (20).

Although, in our pilot study, the sole newborn detected through NBS demonstrated a notable decrease in KREC levels and CD19^+^ cell counts, others have demonstrated T-cell lineage impairment in DS (21).

DS syndrome patients are known to develop many immunological problems such as increased risk of infections, poorer clinical outcomes, and chronic inflammation (14). According to the findings of the current study, newborns with DS exhibit general defects of T-cell and B-cell lineages. Given these observations, it is recommended that all newborns with DS undergo evaluation by an immunologist. Additionally, slightly more than 1% of DS newborns will present with significant lymphopenia, suggesting the necessity for more than just observation; these individuals may require prompt intervention and treatment. On a nationwide scale, this would translate to approximately 11–12 newborns with DS being detected through nationwide newborn screening (NBS) in 2023 (22). Although our data suggests that newborns with DS demonstrate signs of immunological defects, it is important to note that there are data indicating that TREC and KREC levels could potentially be restored later in life in some of them (23).

## 5 Conclusions

Newborns with DS exhibit a decrease in TREC/KREC values that suggests abnormalities of T-and B-cell lineages development and requires further investigation. Given these observations, it is recommended that all newborns with DS undergo evaluation by an immunologist. Slightly more than 1% of DS newborns will present with significant lymphopenia which can be detected with newborn screening measuring both TREC and KREC.

## Data Availability

The datasets presented in this article are not readily available because no new data was generated. Requests to access the datasets should be directed to the corresponding author, marakhonov@generesearch.ru.
